# An Artificial Intelligence Algorithm for Early Detection of Left Ventricular Systolic Dysfunction in Patients with Normal Sinus Rhythm

**DOI:** 10.3390/jcm14124257

**Published:** 2025-06-15

**Authors:** Seongjin Park, Hyo Jin Lee, Sung-Hee Song, KyungChang Woo, Jiwon Kim, Juwon Kim, Ju Youn Kim, Seung-Jung Park, Young Keun On, Kyoung-Min Park

**Affiliations:** 1 Division of Cardiology, Department of Internal Medicine, Heart Vascular Stroke Institute, Samsung Medical Center, Sungkyunkwan University School of Medicine, Seoul 06351, Republic of Korea; 2 Division of Cardiology, Department of Internal Medicine, Samsung Changwon Hospital, Sungkyunkwan University School of Medicine, Changwon 51353, Republic of Korea; 3 Wellysis Co., Seoul 06351, Republic of Korea; 4 MediFarmSoft Company Ltd., Seoul 06351, Republic of Korea; 5 Division of Cardiology, Department of Internal Medicine, Kangbuk Samsung Hospital, Sungkyunkwan University School of Medicine, Seoul 06351, Republic of Korea

**Keywords:** artificial intelligence, electrocardiogram, sinus rhythm, echocardiography, systolic dysfunction

## Abstract

**Background/Objectives:** Most previous studies using artificial intelligence (AI) to detect left ventricular systolic dysfunction (LVSD) from electrocardiograms (ECGs) relied on data obtained near the time of echocardiography or included patients with known cardiac disease, limiting their specificity for screening. We aimed to evaluate whether AI models could predict future LVSD from ECGs interpreted as normal and recorded one to two years before echocardiography. **Methods:** We retrospectively analyzed 24,203 sinus rhythm ECGs from 11,131 patients. Two convolutional neural network models (DenseNet-121 and ResNet-101) were trained (70%), validated (10%), and tested (20%) to predict LVSD (defined as ejection fraction ≤50%). Survival analysis was performed using Kaplan–Meier curves and the log-rank test. **Results:** Of the total population, 2734 patients had LVSD and 8397 had preserved EF. DenseNet-121 and ResNet-101 demonstrated excellent discrimination for LVSD with AUROCs of 0.930 and 0.925, accuracies of 0.887 and 0.860, sensitivities of 0.821 and 0.856, and specificities of 0.908 and 0.861, respectively. In the test set, patients predicted to have LVSD showed a significantly higher risk of echocardiographic LVSD (hazard ratio 9.89, 95% CI 8.20–11.92, *p* = 0.005) and lower 24-month survival (log-rank *p* < 0.001). **Conclusions:** AI-enabled ECG models predicted future LVSD from clinically normal ECGs recorded up to two years prior to imaging. These findings suggest a potential role for AI-ECG in the early detection of subclinical LVSD and improved risk stratification in asymptomatic individuals.

## 1. Introduction

### 1.1. Subclinical Cardiac Dysfunction and Preventive Strategies

Cardiovascular diseases, including heart failure (HF), remain the leading cause of morbidity and mortality worldwide and constitute a major public health burden. To optimize preventive strategies, it is critical to identify individuals with asymptomatic but significant cardiac dysfunction before clinical deterioration, as early myocardial injury may be detectable even in the absence of overt symptoms or acute events [1].

Even in the absence of symptoms, those with left ventricular systolic dysfunction (LVSD) have a 1.7-fold higher death rate and a 4.7-fold increased risk of heart failure compared to the LVSD-free population [2].

Prediction of future LVSD cases has been attempted using biomarkers such as plasma natriuretic peptide [3,4]. However, these attempts did not yield satisfactory results [5,6]. Particularly, methods using biomarkers demonstrated lower sensitivity in the prediction of LVSD occurrence in cases in which ejection fractions (EFs) were ≤50% compared to those in whom EFs were ≤40%. This resulted in having limited success in cases of mild LVSD [7].

### 1.2. Deep Learning-Based Electrocardiographic Analysis for LVSD Detection

The integration of artificial intelligence into cardiology, particularly for diagnostic and prognostic applications such as ECG interpretation, has gained increasing attention in recent years [8]. Studies using artificial intelligence (AI) algorithms to analyze 12-lead electrocardiograms (ECGs) for predicting LVSD have demonstrated significantly better predictive performance, even in prospective validations [9,10,11]. Furthermore, research testing of AI-enhanced predictive performance in evaluating 12-lead ECGs in clinical practice has demonstrated improved diagnostic rates for LVSD [12]. Similar findings have also been observed in studies utilizing single-lead ECGs [13,14]. More recently, a study reported a high negative predictive value in pathological ECGs like those observed in cases of atrial fibrillation (AF) with rapid ventricular response [15].

### 1.3. Study Objective and Clinical Gap

However, most prior work has focused on ECGs acquired near the time of imaging or from patients with overt abnormalities. To our knowledge, no study has evaluated the EKG results of patients determined to have sinus rhythms on ECGs recorded one to two years before echocardiography to test their predictive value for future LVSD occurrence. Thus, the aim of this study was to test the value of the available methods used for predicting the onset of LVSD in patients with sinus rhythms.

## 2. Materials and Methods

### 2.1. Study Population

We conducted a retrospective review of data from adult patients who received transthoracic echocardiography (TTE) from January 2010 to November 2022 at Samsung Medical Center, a single tertiary hospital in Seoul, South Korea. From a total of 344,506 patients with 366,451 ECGs, we identified individuals who had at least one 12-lead ECG documented between one and two years before their index echocardiographic assessments. Patient whose data lacked ECG results within the specified time frame or those in whom the ECGs exhibited results other than sinus rhythm, e.g., AF or paced rhythm, were excluded. We additionally excluded patients whose ECGs were deemed abnormal by either an automated ECG system or a physician’s assessment. The final study cohort consisted of 11,131 patients with 24,203 sinus rhythm ECGs, all of which were interpreted as clinically normal. Each ECG was categorized based on echocardiographic left ventricular EF as either LVSD with EF ≤ 50% or preserved EF with EF > 50%.

This study was approved by the Institutional Review Board of Samsung Medical Center, South Korea (approval date: 6 July 2021; IRB No. 2021-06-088). The requirement for written informed consent was waived due to the retrospective nature of the study.

### 2.2. ECG Acquisition

All ECGs were conventional 10 s, 12-lead recordings obtained at a sampling rate of 500 Hz and digitally archived in the hospital’s ECG management system. Raw ECG waveform data were collected and subjected to pre-processing using amplitude normalization and baseline correction. Only ECGs obtained between 365 and 730 days before echocardiography were included. In cases in which patients had several qualifying ECGs, all ECGs within the specified timeframe were utilized. For patient-level analyses such as survival and Cox regression, only one ECG per patient—the one closest to the echocardiography date—was used.

### 2.3. Algorithm Development

We developed deep learning models based on DenseNet-121 and ResNet-101, both of which are widely used convolutional neural network (CNN) architectures. The input to each model was a 10 s; 500 Hz; and 8-lead ECG waveform—specifically, leads I, II, and V1–V6—structured as a multichannel time series. The dataset was randomly divided into training (70%), validation (10%), and test (20%) sets, and the data were stratified by EF status. The models were trained using the Adam optimizer and determination of binary cross-entropy loss. Early stopping was applied based on validation loss. Each model was trained for up to 20 epochs with a batch size of 128.

### 2.4. Outcomes

Two convolutional neural network models, DenseNet-121 and ResNet-101, were developed to predict LVSD that was defined as an EF ≤ 50% on echocardiography; patient ECGs recorded one to two days prior to imaging were evaluated. These models were trained and tested separately, and their predictive performances were evaluated using the area under the receiver operating characteristic curves (AUCs), sensitivities, specificities, accuracies, and confusion matrices at a predefined probability threshold. Survival outcomes were also evaluated using statistical methods that included Kaplan–Meier estimates and Cox regression analyses. These were based on both echocardiographic EF classification and AI-predicted LVSD status.

### 2.5. Statistical Analysis

The distribution of continuous variables was evaluated using descriptive statistics and a visual inspection of histograms, but formal normality testing was not performed for all variables due to the large sample size. Continuous variable data were summarized using means with standard deviations or medians with interquartile ranges, and categorical variable data were expressed as frequencies and percentages. Each model’s performance was evaluated using AUCs obtained from receiver operating characteristic (ROC) curves. Sensitivities, specificities, positive predictive values (PPVs), and negative predictive values (NPVs) were calculated from the test set using a fixed probability threshold. Cox proportional hazards regression analyses were performed to evaluate the association between model-predicted LVSD and the actual occurrence of LVSD observed in follow-up echocardiography. Kaplan–Meier survival analysis was used to evaluate all-cause mortality between patients with preserved EFs > 50%, and those with LVSD, EFs ≤ 50%, as assessed by echocardiography. Survival distributions were analyzed using the log-rank test, and 95% confidence intervals (CIs) were calculated for each group. Also, Kaplan–Meier analysis was used based on the AI model’s predicted LVSD status using a predefined probability threshold ≥0.10; survival differences were similarly evaluated using the log-rank test. All statistical analyses were conducted using Python (version 3.9.7) and R (version 4.2.2).

### 2.6. Use of Generative AI Tools

During the preparation of the graphical abstract, OpenAI’s ChatGPT (GPT-4, March 2024 version) was used to assist in generating visual concept ideas. The authors reviewed and edited the final output and take full responsibility for the content.

## 3. Results

### 3.1. Baseline Characteristics of the Study Population

**Figure 1:**
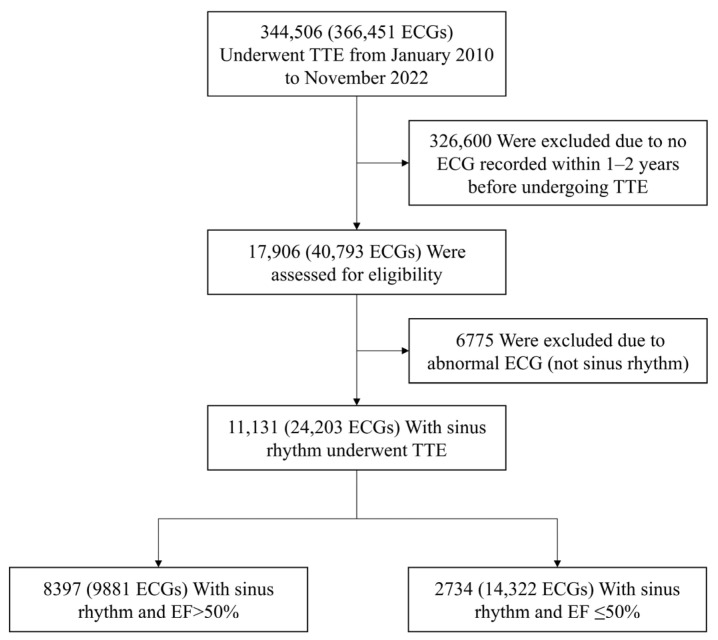
Flowchart of eligible subject selection. Flow diagram showing the selection of eligible patients from the ECG and echocardiography databases. ECGs with non-sinus rhythms or interpretations of being abnormal were excluded. The final cohort included 11,131 patients and 24,203 sinus rhythm ECGs interpreted as normal. ECG, electrocardiogram; TTE, transthoracic echocardiography.

Among the data from 11,131 patients that were included in the final analysis, the data from 8397 patients on whom 9881 ECGs were performed indicated that these patients had preserved left ventricular EFs, 50%. Data from 2734 patients on whom 14,322 ECGs were performed indicated the occurrence of LVSD, defined as EF ≤ 50%, based on echocardiographic assessments performed one to two years after the index ECGs (Figure 1). Patients with LVSD were older (mean 67.8 ± 12.1 years vs. 56.2 ± 11.3 years) and more likely to be male (68.4% vs. 51.4%) than those with preserved EF (Table 1). Sex data were unavailable for a small proportion of patients.

### 3.2. Model Performance

**Figure 2:**
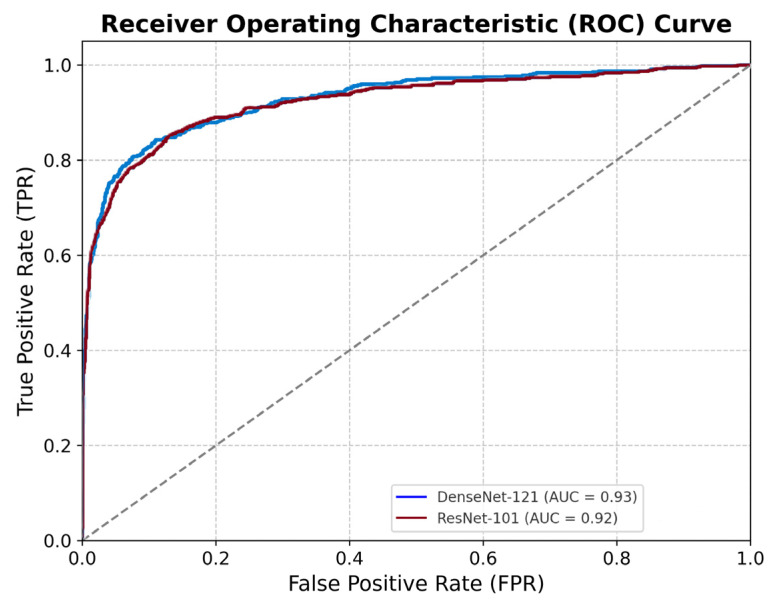
ROC curves of AI models predicting LVSD. The ROC curves of the primary and secondary convolutional neural network models for predicting LVSD, EF ≤ 50%, from sinus rhythm ECGs acquired one to two years prior to echocardiography performance are shown. The AUCs were 0.930 and 0.925 for the two models. The diagonal dotted line represents the line of no discrimination (AUC = 0.5), indicating random performance. ECG, electrocardiogram; ROC, receiver operating characteristic; LVSD, left ventricular systolic dysfunction; AUC, area under the curve.

**Figure 3:**
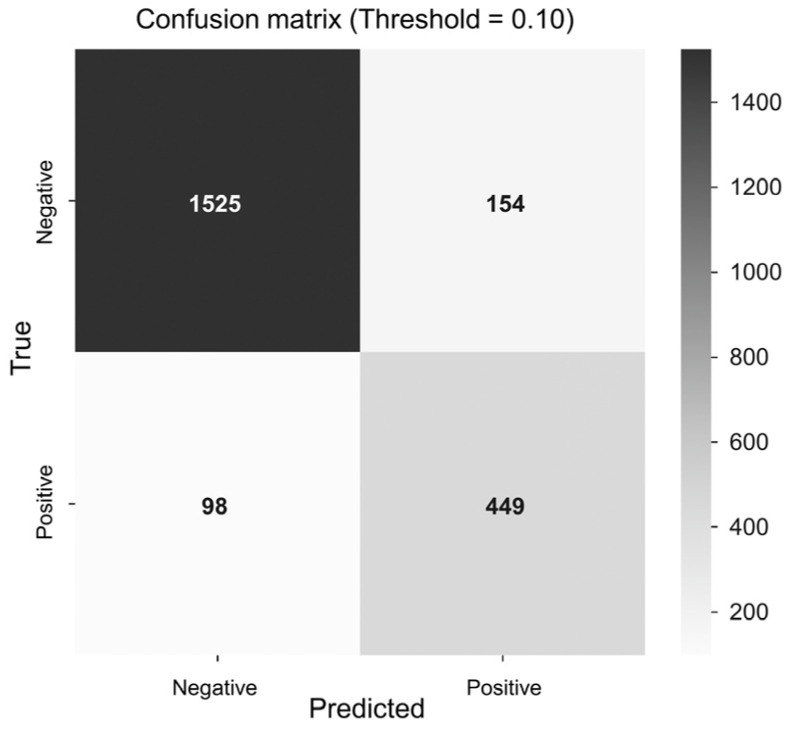
Confusion matrices for AI prediction of LVSD. Confusion matrices for the test set were based on a predefined threshold of ≥0.10 for both models. Sensitivity, specificity, and accuracy are displayed for each model. AI, artificial intelligence; LVSD, left ven-tricular systolic dysfunction.

Both CNN models demonstrated excellent predictive performance in identifying LVSD from sinus rhythm ECGs recorded one to two years prior to echocardiography. The DenseNet-121 model achieved an AUC of 0.930, a sensitivity of 0.821, a specificity of 0.908, and an overall accuracy of 0.887. The ResNet-101 model achieved comparable results: an AUC of 0.925, a sensitivity of 0.856, a specificity of 0.861, and an accuracy of 0.860 (Figure 2 and Figure 3).

### 3.3. Association with Echocardiographic LVSD

Among patients in the test set, those identified by either AI model as likely to develop LVSD (model output ≥ 0.10) were significantly more likely to have LVSD on echocardiography compared to those predicted to have preserved EF. The hazard ratio for echocardiographic LVSD in the AI-predicted positive group was 9.89 with a 95% CI of 8.20–11.92 and *p*-value of 0.005.

### 3.4. Survival Outcomes Based on Echocardiographic and AI Classification

**Figure 4:**
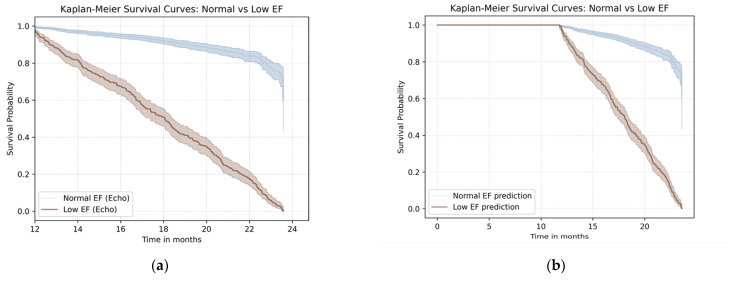
Kaplan–Meier survival curves according to echocardiographically determined ejection fraction status and AI-predicted LVSD. (**a**) Kaplan–Meier curves comparing overall survival between patients with preserved ejection fraction (EF > 50%) and those with LV systolic dysfunction (EF ≤ 50%) as determined by echocardiography; (**b**) Kaplan–Meier analysis comparing all-cause mortality between patients predicted to have LVSD (model output ≥ 0.10) and those not predicted to have LVSD by the AI model. In both panels, patients classified as having LVSD showed significantly reduced survival over the 24-month follow-up period. Shaded areas indicate 95% confidence intervals. EF, ejection fraction; LV, left ventricle; AI, artificial intelligence; LVSD, left ventricular systolic dysfunction.

Kaplan–Meier survival analysis revealed that patients with LVSD, EF ≤ 50%, had a significantly lower all-cause survival rate over a 24-month follow-up period compared to those with preserved EF (log-rank *p* < 0.001) (Figure 4a).

Similarly, patients predicted by the AI model to have LVSD (model output ≥ 0.10) also demonstrated a significantly reduced survival rate compared to those predicted to have preserved EFs. The hazard ratio for all-cause mortality in the AI-predicted LVSD group was 3.24 with a 95% CI of 2.72–3.86 and a log-rank *p* < 0.001 (Figure 4b).

## 4. Discussion

### 4.1. Early Prediction of LVSD Using AI-Enabled ECG

Previous studies have demonstrated the feasibility of detecting LVSD using artificial intelligence (AI)-enabled analysis of 12-lead ECGs. The model developed by Attia et al. achieved an AUC of 0.93 in predicting EFs ≤ 35% using ECGs recorded near the time of echocardiography, and subsequent studies validated similar models in various settings including emergency departments [16]. However, most of these studies analyzed ECGs performed concurrently with imaging and often included patients with overtly abnormal ECGs or clinical symptoms.

Our study, however, focused on a distinct clinical situation for which research data are lacking. This situation is the possibility of predicting LVSD development from normal sinus rhythm EKGs recorded 1–2 years prior to imaging. Our results confirm that a method is available for accomplishing this task. The AI model that we tested demonstrated excellent performance in predicting future LVSD occurrences from ECGs that were not only temporally remote from the performance of echocardiography but also devoid of overt abnormalities. This proved the method’s capability to identify subtle subclinical electrophysiologic features that precede structural cardiac dysfunction.

Additionally, the clinical validity of the AI-based risk classification system was substantiated by the significant correlation between the model’s predictions and actual echocardiographically confirmed LVSD development during follow-up (hazard ratio = 9.89 and 95% CI = 8.20–11.92) [17,18].

### 4.2. AI-ECG and Subclinical Myocardial Remodeling

The mechanisms underlying the ability of AI to detect early LVSD have not been completely elucidated. However, recent studies suggest that machine learning models can extract subtle temporal and morphological patterns from ECGs that are otherwise imperceptible [19]. These patterns may reflect subclinical myocardial remodeling, such as changes in conduction velocity, repolarization dispersion, or inter-lead electrical relationships, that precede overt structural dysfunction. The model’s capacity to detect at-risk patients despite unremarkable normal sinus rhythm ECG findings confirms that AI can identify early electrophysiological indicators of ventricular dysfunction [9,18]. These risk classifications were associated with the ability to predict future electrocardiographically confirmed LVSD diagnoses and a 24-month survival rate. This demonstrates the method’s prognostic value (Figure 4b).

These capabilities were also observed in an atrial arrhythmia study. Christopoulos et al. established that an AI-ECG model, trained on sinus rhythm recordings, could forecast the onset of AF years prior to its development; more than 50% of the high-risk group developed AF within 10 years. Their model performed comparably to the CHARGE-AF risk score but required no clinical data [20].

Together, these findings demonstrate the clinical utility of AI-ECG in functioning as an efficient digital biomarker in identifying preclinical electrical alterations that occur in certain cardiovascular disorders.

### 4.3. Clinical Implications of AI-ECG for Population Screening

Our findings suggest that our AI-enabled ECG method would be effective in screening for asymptomatic LVSD. Because ECGs are widely performed in general practice, health follow-up examinations, and even preoperative evaluations, the integration of AI-based interpretation into routine ECG monitoring systems could allow for timely risk stratification and early referral for echocardiographic assessment. This integration may be particularly beneficial in resource-constrained medical facilities or among individuals at elevated risk of cardiomyopathy development, e.g., individuals with diabetes, hypertension, or cancer survivors following anthracycline treatment. Early identification of preclinical LVSD would enable timely therapeutic intervention, potentially preventing progression to symptomatic heart failure. Recent studies have established the cost-effectiveness of AI-ECG-based screening for LVSD. Liu et al. discovered that opportunistic screening utilizing AI-ECG could result in cost reductions, especially when utilized for older patients [21]. Similarly, an analysis of cost-effectiveness by Tseng et al. estimated an incremental cost-effectiveness ratio of $43,351 per quality-adjusted life year for universal AI-ECG screening at age 65 to detect asymptomatic LVSD. Therefore, this screening strategy is cost-effective in most clinical situations [22].

More recently, Dhingra et al. developed and validated an image-based AI-ECG model in three international cohorts (YNHHS (New Haven, CN, USA), UK Biobank (Manchester, UK), and ELSA-Brasil (Campinas, Brazil)). This research group demonstrated that a positive screen was associated with a four- to twenty-four-fold increased risk of heart failure. Their research findings corroborate those that demonstrate the ability of ECGs, even 12-lead EKGs, to function as digital biomarkers for the potential development of heart failure with substantial efficacy, irrespective of the design of the ECG apparatus or its formatting. These results reinforce the generalizability and feasibility of clinical AI-ECG screening strategies for structural heart disease. Their analysis focused on future heart failure events in a general population. Our study results complement their findings by showing that the excellent AI prediction of LVSD capabilities using normal sinus rhythm ECGs were associated with both structural dysfunction and adverse survival outcomes [23].

These findings align with an increasing interest in the use of pre-emptive therapies in high-risk populations and emphasize the evolving role of AI in cardiovascular disease prevention [17]. Importantly, our study demonstrated markedly lower electrocardiographically confirmed and AI-predicted survival rates than the rates for those with preserved EF (Figure 4a), reaffirming the clinical importance of early detection and the clinical relevance of both structural and electrophysiological markers in identifying patients at risk (Figure 4). AI-ECG can identify individuals at risk for multiple subclinical cardiovascular diseases including systolic dysfunction and AF [20].

### 4.4. Limitations

This study had several limitations. First, this was a single-center retrospective study with the limitations inherent to this type of study, including limited generalizability and the possible presence of selection bias and unidentified confounding factors. Although the AI model was developed and evaluated using a single institutional dataset, the inclusion of over 11,000 patients from both health screening and clinical populations across varied acquisition settings adds significant internal heterogeneity and enhances the real-world applicability of our findings. Furthermore, the inclusion of normal sinus rhythm ECGs obtained 1–2 years prior to echocardiography from an asymptomatic population reflects a clinically relevant time window, adding novelty and real-world applicability to our findings. Nonetheless, external validation across population, equipment, and workflow variations will be critical to ensure generalizability.

Moreover, detailed clinical variables such as smoking status and comorbidities (e.g., hypertension, diabetes, and cardiovascular disease) were not available for inclusion in the analysis due to limitations in the retrospective dataset. This lack of information limited our ability to adjust for important confounding factors, potentially affecting the interpretation of the model’s predictive performance and prognostic implications. However, our large sample size and long follow-up period may partially mitigate these limitations by improving the stability of the observed associations.

In addition, although we excluded ECGs with overt abnormalities, our criteria relied on clinical ECG interpretations rather than formal re-annotations by electrophysiologists. Nevertheless, this pragmatic approach reflects routine clinical workflow, thereby enhancing the relevance and applicability of our findings to real-world practice.

We did not stratify patients by EF severity, e.g., mid-range vs. reduced EF, or examine outcomes such as hospitalization for heart failure or mortality. Additionally, cause-specific mortality data were not available. This limited our ability to verify that the observed prognostic differences were cardiovascular in origin.

The AI output threshold (0.10) was chosen empirically and not optimized through formal calibration. Future studies should evaluate threshold selection strategies for clinical deployment.

We also acknowledge that interpretability remains a critical challenge in the clinical deployment of deep learning models. Due to the unavailability of the original modeling environment and architecture, we could not perform saliency map or Grad-CAM visualizations. Future studies incorporating such interpretability techniques are essential to improve clinical transparency and user trust.

Finally, while our Kaplan–Meier analyses demonstrated prognostic separation based on both echocardiographically and AI-derived classifications, further investigation is warranted to determine the validity of the assertion that early AI-guided identification results in improved clinical outcomes. Studies that assess multivariable models that adjust for traditional risk factors are needed to certify that AI-predicted LVSD serves as an independent prognostic marker.

## 5. Conclusions

In this study, we demonstrated that a deep learning model applied to sinus rhythm ECGs that were interpreted as normal and recorded one to two years prior to echocardiography performance can effectively predict the future development of LVSD. This finding suggests that subtle electrical changes associated with early myocardial remodeling may be promptly detectable by AI, well before overt structural dysfunction is apparent on imaging.

Our results emphasize that AI-enabled ECG analysis has the potential to be a non-invasive, widely accessible tool for the early identification of patients at risk for LVSD who are without symptoms or abnormal ECG findings. If validated in prospective and multicenter studies, this approach may offer a valuable strategy for screening and early intervention in asymptomatic populations.

## Figures and Tables

**Table 1:** Baseline characteristics of patients with preserved EF and LVSD.

	Number of Patients	ECG No.	Age, Years	Male (%)	Female (%)	Unknown Sex (%)
Normal EF (EF > 50%)						
Total population	8397	9582	56.2 ± 11.3	4316 (51.4)	3776 (45.0)	305 (3.6)
Training group (70%)	5877	6906	56.3 ± 11.2	3025 (51.5)	2631 (44.8)	221 (3.8)
Validation group (10%)	840	996	55.2 ± 11.4	417 (49.6)	390 (46.4)	33 (3.9)
Test group (20%)	1680	1680	56.2 ± 11.5	874 (52.0)	755 (44.9)	51 (3.0)
LVSD (EF ≤ 50%)						
Total population	2734	14,322	67.8 ± 12.1	1870 (68.4)	722 (26.4)	142 (5.2)
Training group (70%)	1913	10,115	62.5 ± 13.1	1314 (68.7)	501 (26.2)	98 (5.1)
Validation group (10%)	274	1298	55.2 ± 11.4	196 (71.5)	63 (23.0)	15 (5.5)
Test group (20%)	547	2909	56.2 ± 11.5	360 (65.8)	158 (28.9)	29 (5.3)

Values are presented as means ± standard deviations or numbers (percentages). Training, validation, and test sets were derived using stratified random sampling in a 70:10:20 ratio. Sex percentages do not sum to 100% because of a small number of patients for whom these data were unavailable. ECG, electrocardiogram; EF, ejection fraction.

## Data Availability

The datasets generated and/or analyzed during the current study are available from the corresponding author on reasonable request. The data are not publicly available due to institutional and ethical restrictions.

## Abbreviations

The following abbreviations are used in this manuscript:

AI	Artificial intelligence
LVSD	Left ventricular systolic dysfunction
ECG	Electrocardiogram
EF	Ejection fraction
CI	Confidence interval
AF	Atrial fibrillation
TTE	Transthoracic echocardiography
CNN	Convolutional neural network
AUC	Area under curve
ROC	Receiver operating characteristic
PPV	Positive predictive value
NPV	Negative predictive value

## References

[1] Jakubiak G.K. (2024). Cardiac Troponin Serum Concentration Measurement Is Useful Not Only in the Diagnosis of Acute Cardiovascular Events. J. Pers. Med..

[2] Wang T.J., Evans J.C., Benjamin E.J., Levy D., LeRoy E.C., Vasan R.S. (2003). Natural history of asymptomatic left ventricular systolic dysfunction in the community. Circulation.

[3] McDonagh T.A., McDonald K., Maisel A.S. (2008). Screening for asymptomatic left ventricular dysfunction using B-type natriuretic Peptide. Congest. Heart Fail..

[4] Vasan R.S., Benjamin E.J., Larson M.G., Leip E.P., Wang T.J., Wilson P.W., Levy D. (2002). Plasma natriuretic peptides for community screening for left ventricular hypertrophy and systolic dysfunction: The Framingham heart study. Jama.

[5] Goetze J.P., Mogelvang R., Maage L., Scharling H., Schnohr P., Sogaard P., Rehfeld J.F., Jensen J.S. (2006). Plasma pro-B-type natriuretic peptide in the general population: Screening for left ventricular hypertrophy and systolic dysfunction. Eur. Heart J..

[6] Redfield M.M., Rodeheffer R.J., Jacobsen S.J., Mahoney D.W., Bailey K.R., Burnett J.C. (2004). Plasma brain natriuretic peptide to detect preclinical ventricular systolic or diastolic dysfunction: A community-based study. Circulation.

[7] Macheret F., Boerrigter G., McKie P., Costello-Boerrigter L., Lahr B., Heublein D., Sandberg S., Ikeda Y., Cataliotti A., Bailey K. (2011). Pro-B-type natriuretic peptide_1–108_ circulates in the general community: Plasma determinants and detection of left ventricular dysfunction. J. Am. Coll. Cardiol..

[8] Cersosimo A., Zito E., Pierucci N., Matteucci A., La Fazia V.M. (2025). A Talk with ChatGPT: The Role of Artificial Intelligence in Shaping the Future of Cardiology and Electrophysiology. J. Pers. Med..

[9] Attia Z.I., Kapa S., Lopez-Jimenez F., McKie P.M., Ladewig D.J., Satam G., Pellikka P.A., Enriquez-Sarano M., Noseworthy P.A., Munger T.M. (2019). Screening for cardiac contractile dysfunction using an artificial intelligence–enabled electrocardiogram. Nat. Med..

[10] Attia Z.I., Kapa S., Yao X., Lopez-Jimenez F., Mohan T.L., Pellikka P.A., Carter R.E., Shah N.D., Friedman P.A., Noseworthy P.A. (2019). Prospective validation of a deep learning electrocardiogram algorithm for the detection of left ventricular systolic dysfunction. J. Cardiovasc. Electrophysiol..

[11] König S., Hohenstein S., Nitsche A., Pellissier V., Leiner J., Stellmacher L., Hindricks G., Bollmann A. (2023). Artificial intelligence-based identification of left ventricular systolic dysfunction from 12-lead electrocardiograms: External validation and advanced application of an existing model. Eur. Heart J.-Digit. Health.

[12] Yao X., Rushlow D.R., Inselman J.W., McCoy R.G., Thacher T.D., Behnken E.M., Bernard M.E., Rosas S.L., Akfaly A., Misra A. (2021). Artificial intelligence–enabled electrocardiograms for identification of patients with low ejection fraction: A pragmatic, randomized clinical trial. Nat. Med..

[13] Attia Z.I., Dugan J., Rideout A., Maidens J.N., Venkatraman S., Guo L., Noseworthy P.A., Pellikka P.A., Pham S.L., Kapa S. (2022). Automated detection of low ejection fraction from a one-lead electrocardiogram: Application of an AI algorithm to an electrocardiogram-enabled Digital Stethoscope. Eur. Heart J.-Digit. Health.

[14] Khunte A., Sangha V., Oikonomou E.K., Dhingra L.S., Aminorroaya A., Mortazavi B.J., Coppi A., Brandt C.A., Krumholz H.M., Khera R. (2023). Detection of left ventricular systolic dysfunction from single-lead electrocardiography adapted for portable and wearable devices. Npj Digit. Med..

[15] Jeong J.H., Kang S., Lee H.S., Lee M.S., Son J.M., Kwon J.-m., Lee H.S., Choi Y.Y., Kim S.R., Cho D.-H. (2024). Deep learning algorithm for predicting left ventricular systolic dysfunction in atrial fibrillation with rapid ventricular response. Eur. Heart J.-Digit. Health.

[16] Adedinsewo D., Carter R.E., Attia Z., Johnson P., Kashou A.H., Dugan J.L., Albus M., Sheele J.M., Bellolio F., Friedman P.A. (2020). Artificial Intelligence-Enabled ECG Algorithm to Identify Patients with Left Ventricular Systolic Dysfunction Presenting to the Emergency Department with Dyspnea. Circ. Arrhythm. Electrophysiol..

[17] Kashou A.H., Medina-Inojosa J.R., Noseworthy P.A., Rodeheffer R.J., Lopez-Jimenez F., Attia I.Z., Kapa S., Scott C.G., Lee A.T., Friedman P.A. (2021). Artificial Intelligence-Augmented Electrocardiogram Detection of Left Ventricular Systolic Dysfunction in the General Population. Mayo Clin. Proc..

[18] Sangha V., Nargesi A.A., Dhingra L.S., Khunte A., Mortazavi B.J., Ribeiro A.H., Banina E., Adeola O., Garg N., Brandt C.A. (2023). Detection of Left Ventricular Systolic Dysfunction from Electrocardiographic Images. Circulation.

[19] Attia Z.I., Harmon D.M., Behr E.R., Friedman P.A. (2021). Application of artificial intelligence to the electrocardiogram. Eur. Heart J..

[20] Christopoulos G., Graff-Radford J., Lopez C.L., Yao X., Attia Z.I., Rabinstein A.A., Petersen R.C., Knopman D.S., Mielke M.M., Kremers W. (2020). Artificial Intelligence-Electrocardiography to Predict Incident Atrial Fibrillation: A Population-Based Study. Circ. Arrhythmia Electrophysiol..

[21] Liu W.T., Hsieh P.H., Lin C.S., Fang W.H., Wang C.H., Tsai C.S., Hung Y.J., Hsieh C.B., Lin C., Tsai D.J. (2024). Opportunistic Screening for Asymptomatic Left Ventricular Dysfunction With the Use of Electrocardiographic Artificial Intelligence: A Cost-Effectiveness Approach. Can. J. Cardiol..

[22] Tseng A.S., Thao V., Borah B.J., Attia I.Z., Medina Inojosa J., Kapa S., Carter R.E., Friedman P.A., Lopez-Jimenez F., Yao X. (2021). Cost Effectiveness of an Electrocardiographic Deep Learning Algorithm to Detect Asymptomatic Left Ventricular Dysfunction. Mayo Clin. Proc..

[23] Dhingra L.S., Aminorroaya A., Sangha V., Pedroso A.F., Asselbergs F.W., Brant L.C.C., Barreto S.M., Ribeiro A.L.P., Krumholz H.M., Oikonomou E.K. (2025). Heart failure risk stratification using artificial intelligence applied to electrocardiogram images: A multinational study. Eur. Heart J..

